# Surface Geometry of Four Conventional Nanohybrid Resin-Based Composites and Four Regular Viscosity Bulk Fill Resin-Based Composites after Two-Step Polishing Procedure

**DOI:** 10.1155/2020/6203053

**Published:** 2020-08-12

**Authors:** Mateusz Granat, Janusz Cieloszyk, Urszula Kowalska, Jadwiga Buczkowska-Radlińska, Ryta Łagocka

**Affiliations:** ^1^Department of Conservative Dentistry and Endodontics, Pomeranian Medical University, Szczecin 70-111, Poland; ^2^Department of Manufacturing Technology, West Pomeranian University of Technology, Szczecin 70-313, Poland; ^3^Center of Bioimmobilisation and Innovative Packaging Materials, West Pomeranian University of Technology, Szczecin 71-270, Poland

## Abstract

**Objectives:**

The aim of the study was to determine the quantitative and qualitative surface structure of contemporary RBCs in posterior teeth reconstructions: regular viscosity bulk fill and conventional composites, obtained after two-stage polishing procedure.

**Materials and Methods:**

Four conventional nanohybrid composites (Tetric EvoCeram, GrandioSO, Filtek Z550, and Ceram·X Mono) and four regular viscosity bulk fill composites (Tetric EvoCeram Bulk Fill, X-tra fil, Filtek Bulk Fill Posterior, and QuixFil) were tested. Samples of each RBC were prepared using PMMA cylindrical mold. After two-step polishing procedure, a surface geometry was evaluated under profilometry (Turbowave v. 7.36, Hommel-Etamic) and SEM (VEGA 3, Tescan Analytics). To evaluate differences between values, the following nonparametric tests were used: Friedman's ANOVA, Wilcoxon's matched-pair test, ANOVA Kruskal-Wallis, and Mann-Whitney *U*.

**Results:**

All conventional RBCs showed Ra values in the range of 0.20-0.26 *μ*m. Bulk fill showed higher values in range of 0.49-1.36 *μ*m except for Filtek Bulk Fill Posterior, which achieved 0.23 *μ*m Ra value. SEM images of conventional RBCs were described as smooth surfaces with slight damage except for TEC, which presented smooth surface with no damage. Bulk fill composites showed rough surface, except for TBF, which presented smooth surface with slight damage.

**Conclusions:**

Regular viscosity bulk fill composites do not constitute a homogeneous group regarding surface roughness after polishing. They obtain, for the most part, poorer smoothness values after polishing than conventional RBCs.

## 1. Introduction

Finishing and polishing of resin-based composites (RBCs) is a necessary procedure which allows to obtain high-quality and esthetic filling [1–10]. According to Jefferies, this step should consist of three stages: (1) gross contouring of the restoration to obtain the desired anatomy, (2) the reduction and smoothing of the surface roughness and scratches created by finishing instruments in the process of gross reduction and initial polishing, and (3) the process of producing a highly smooth, light-reflective, enamel-like surface through final polishing [1]. Composite polishing is a difficult procedure because of heterogeneous nature of composite resin, i.e., tough particles of inorganic filler embedded in a relatively soft organic matrix [3, 4, 6]. Roughness of RBC surface depends on type and properties of polishing instrument as well as structure and properties of the composite material and interactions between them [2–5, 9–11]. According to St-Pierre et al., material-dependent factors that affect surface roughness are (1) the resin matrix content and formulation, (2) the filler particle characteristics (type, hardness compared with the abrasiveness of the polishers, size, and shape), (3) the composite resin filler load, (4) the quality of the silane coupling agent, and (4) the degree of conversion after light curing [10]. Roughness of composite surface is mainly dictated by size, hardness, shape, and quantity of inorganic filler. Bigger filler particles correspond with more rough composite surfaces after polishing procedures [3–5, 9–14]. Differences in sizes and arrangement of filler particles cause different composite materials to have various roughness of surface using the same polishing technique. The physical state of the surface of the RBC can be measured as surface roughness. Surface roughness, given as the roughness value—Ra, does not present sufficient information about gloss or tooth appearance after wetting, because subjective feelings depend on many other factors [15]. This value however corresponds with smoothness which is responsible for dental plaque accumulation, discoloration of material surface and as a consequence risk of secondary caries, and inflammation of marginal gingiva [4, 5, 8–12, 16, 17]. Bollen et al. proved that surface roughness at the level 0.2 *μ*m is sufficient to reduce adhesion of bacteria to the filling surface [18]. According to Chung, composite surface seems to be optically smooth when Ra value is below 1 *μ*m [19]. Willems et al. stated that final roughness of dental composite should be similar to enamel roughness in occlusal contact points, which is about 0.64 *μ*m [20]. These data are in conflict with several studies which reported that there were no differences in plaque accumulation throughout the roughness (Ra) range of 0.7 − 1.4 *μ*m [16, 21]. Roughness comparative studies of novel composite materials, nanofills, suprananofills, nanohybrid, and microhybrid after one and multistep finishing and polishing procedures, do not clearly determine which material and polishing system brings the best results [2–10, 14, 22]. However, they confirm that roughness of composite structure depends on both the polishing system used and composite material structure and composition [4–6]. Last decade brought the need of finishing and polishing bulk fill composites—novel materials with modified structure of organic matrix and inorganic fillers. In contrast to conventional composites, which require incremental placement, these materials contain more sensitive photoinitiators that allow the depth of cure to reach up to 4-5 mm while maintaining predictable degree of conversion [23, 24]. This would allow dentists to place a single increment in deep lesions without the need for a layering technique expediting the restorative procedure and decreasing the overall chair time [24, 25]. Even though bulk fill formulations are mainly considered for posterior applications, maintaining basic esthetic characteristics of the resin is required. Surface roughness could affect the plaque accumulation and color stability which could affect the survival of composite restorations as well as the dentist's decision for replacement. Bulk fill composite materials, due to the need of polymerization shrinkage reduction, often contain large, irregular filler particles in their structure, which can suggest worse polishability compared to conventional composites. However, studies comparing the roughness of bulk fill and conventional composites are very limited [5, 11, 30, 31].

The aim of the study was to determine the quantitative and qualitative surface structure of contemporary RBC used in posterior teeth reconstruction: regular viscosity bulk fill and conventional resin based composites, obtained after a two-step polishing procedure. The following null hypotheses were made: H01—there will be no differences of obtained surface roughness values and surface SEM images within the group of conventional RBC and bulk fill RBCs; H02—there will be no differences of the surface roughness values and surface SEM images between bulk fill and conventional RBC.

## 2. Materials and Methods

Eight RBC with different structures of inorganic and organic matrix were evaluated. Four of them were conventional nanohybrid RBCs: Tetric EvoCeram (Ivoclar Vivadent, Schaan, Liechtenstein), GrandioSO (VOCO, Cuxhaven, Germany), Filtek Z550 (3M-ESPE, St. Paul, USA), and Ceram·X Mono (Dentsply, Konstanz, German), and the other four were regular viscosity bulk fill composites: Tetric EvoCeram Bulk Fill (Ivoclar Vivadent, Schaan, Liechtenstein), X-tra fil (VOCO, Cuxhaven, Germany), Filtek Bulk Fill Posterior (3M-ESPE, St. Paul, USA), and QuixFil (Dentsply, Konstanz, Germany). A detailed description of the materials is provided in Table 1.

### 2.1. Preparation of Specimens

Unpolymerized RBC was placed in PMMA cylindrical mold with 10 mm diameter and 2 mm height for conventional composites and 4 mm height for bulk fill ones. Mold was filled with material placed in one portion; then, it was covered with a 50 *μ*m thick polyester strip according to ISO 4049: 2009 [26]. Polyester strip was covered with a glass microscopic plate, and 2 kg weight was applied for 30 seconds to remove excess material. Specimens were then polymerized for 20 seconds with 1470 mW/cm^2^ (-10%/+20%) curing light (3M™ Elipar™ DeepCure-S LED Curing Unit). The light intensity of the curing unit was measured using a manual radiometer (Spring 2K Light Meter, SPR-SP3K, Spring Health Products, Inc., Morristown, PA). Directly after polymerization, specimens were polished using low-speed handpiece WE-56 LED G (W&H Dentalwerk Bürmoos GmbH) with two-step polishing system Politip (Ivoclar Vivadent, Schaan, Liechtenstein). The procedure was carried out according to the manufacturer's recommendations at a speed of 8000 rpm, with water cooling 50 ml/min. The shape of the polishers was a small cup. Each specimen was polished with a new set of polishers. The procedure was carried out by one operator, using average pressure 2 N for 30 seconds. Measurement was made by putting a specimen onto laboratory scale. After taring the weight, polishing was started not exceeding the value of 200 g. Then, the samples were placed in distilled water and stored at 37°C. Six samples of each material were made, which were randomly divided into two groups: five for profilometry and one for SEM examination.

### 2.2. Profilometric Examination

Graphic presentation of the tested surface profile of the composite material, registered (P), waviness (W), and roughness (Ra), was made using a Hommel-Etamic Turbowave v. 7.36 profilometer using a TK100 measuring tip. After stabilization of the sample in the profilometer, the roughness of the composite resin surface was measured by sliding the measuring head of the profilometer along the tested surface at a measuring speed of 0.50 mm/s using an ISO 11562 filter. Each surface was scanned five times along 4.8 mm parallel sections. The profilometric tests were carried out at the Department of Machine Technology of the West Pomeranian University of Technology in Szczecin.

### 2.3. SEM Examination

Surface micromorphology of composite material samples was imaged using a VEGA 3 scanning electron microscope (Tescan Analytics, Fuveau, France). Prior to imaging, the samples were placed in a vacuum sputter with rotary pump Q150R (Quorum Technologies Ltd., Laughton, United Kingdom) and sputtered with gold to ensure appropriate sample conduction. The materials were tested at an accelerating voltage of 30 kV and an SE secondary electron detector. SEM images were captured at 1000x and 3000x magnification. The tests were carried out at the Center of Bioimmobilisation and Innovative Packaging Materials of the West Pomeranian University of Technology in Szczecin.

The imaged surface of the materials was assessed descriptively. Photoprints 12 × 12 cm in size were used. Each image was divided into 16 equal square boxes, with each square being assessed separately with respect to surface roughness, using four gradings: 0: homogeneous surface, without damage; 1: presence of surface defects (fractures, cracks, scratches); 2: presence of filler particles not surrounded by the matrix; 3: the presence of places in the matrix from which the filler particles were removed.

After adding up the results from all fields, a qualitative assessment of the material surface was made according to the values: 0-12: smooth surface, no damage; 12-24: smooth surface with slight damage; 24-36: rough surface; 36-48: rough surface with significant damage.

**Figure 1:**
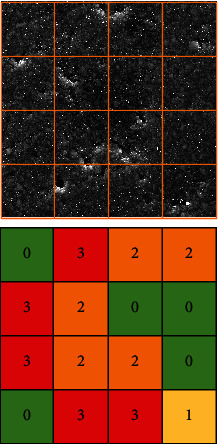
Example descriptive assessment of material surface roughness based on SEM image. The material obtained 24 points, so its surface was considered smooth, with slight damage.

During the SEM examination, the type of composite was blind. After calibration in qualitative evaluation of roughness, assessment of the photomicrographs was carried out by two individuals. Kappa Cohen's test was used to assess the examiners compliance. Substantial within-rater reliability was achieved *κ*_w_ = 0.68–0.97. The total measure within-rater and interrater reliability was *κ*_win_ = 0.70. Example description of surface condition is shown on Figure 1.

### 2.4. Statistical Analysis

Statistical analysis was performed using STATISTICA for Windows 9.0 (StatSoft, Inc.). To evaluate differences between values, the following nonparametric tests were used: Friedman's ANOVA, Wilcoxon's matched-pair test, ANOVA Kruskal-Wallis, and Mann-Whitney *U*. A probability of less than 0.05 was considered significant, and below 0.01 was considered highly significant.

## 3. Results

**Figure 2:**
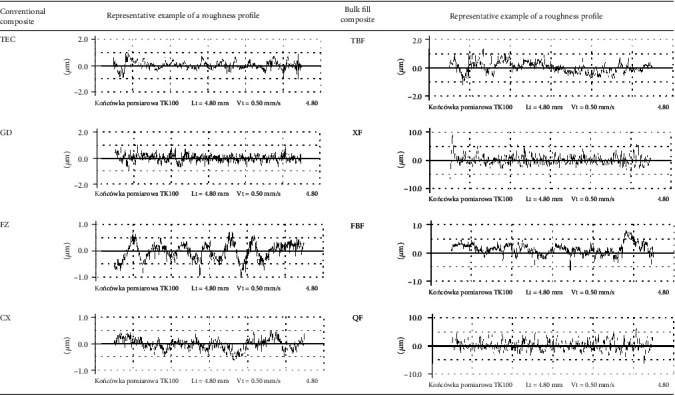
Sample profilometry plots of RBCs used in the study. LT: traversing length; Vt: traversing speed; TK100: measuring tip.

The average values of tested materials' surface roughness after polishing are presented in Table 2. Tested composites showed different values of surface roughness. All conventional composite materials (TEC, GD, FZ, and CX), polymerized in a 2 mm layer, showed Ra in the range of 0.20-0.26 *μ*m. Bulk fill materials (TBF, XF, QF), polymerized in 4 mm layers, after polishing, obtained several times higher Ra values (TBF < XF, QF) than conventional materials, except for the FBF material for which the average Ra coefficient was 0.23 *μ*m. Representative images of surface roughness profiles of the tested RBCs are shown in Figure 2.

**Figure 3:**
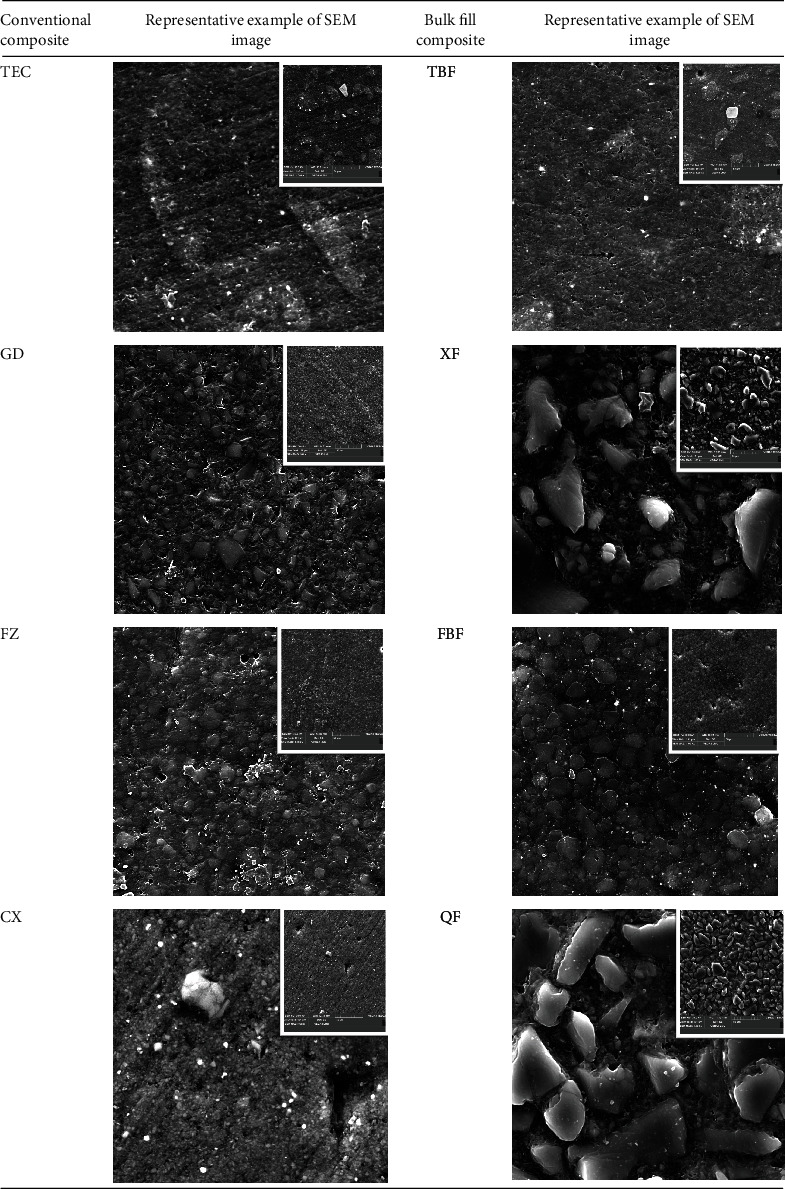
SEM images of RBCs used in study. Magnification 3000x, right upper corner 1000x.

SEM images of polished material surfaces are presented in Figure 3. SEM image of the material surfaces (GD, FZ, CX) after two-step polishing shows similar, homogeneous surfaces, with visible filler particles with similar size. Only in the case of TEC material, apart from very fine filler particles, larger irregular particles with dimensions from 30-70 *μ*m are also visible. SEM images of the regular viscosity bulk fill RBCs surfaces are diverse. Filtek Bulk Fill shows a homogeneous surface close in appearance to conventional materials: GD, CX, and similiar to FZ. The SEM image of Tetric EvoCeram Bulk Fill is similar to the image of Tetric EvoCeram, but with less frequently spaced large and irregular filler particles among very fine ones. SEM images of QF and XF surfaces show a mixture of medium and large irregular molecules with different sizes clearly protruding above the surrounding matrix and fine inorganic filler particles. The obtained SEM images of QF and XF surfaces correspond to the surface roughness values of these materials. The introduced own point scale describing SEM images confirmed the surface condition of the materials (Table 3). The surface of all conventional composites can be described as a smooth surface (TEC) or a smooth surface with slight damage (GD, FZ, and CX). Only TBF surface from bulk fill materials can be described as smooth with slight damage. The surface of FBF, QF, and XF was described as a rough surface.

## 4. Discussion

Finishing of RBC surface with rotating devices is necessary to remove any excess of material and reduce possible excess contacts in occlusion [1]. Most restorations need final contouring and polishing to obtain a smooth and high gloss surface imitating a natural tooth. According to Pereira et al., polyester strip promotes greater smooth surface to composite restoration, but clinically, it is rarely used [27]. There are many dental finishing and polishing methods for obtaining good surface quality [1, 28]. Most studies state that the smoothest surface can be obtained after using a single polishing system with multistep like SuperSnap (Shofu, Inc., Kyoto, Japan) or Sof Lex (3M ESPE) [6, 9, 10, 28, 29].

In this study, since RBCs used to fill cavities in the posterior teeth were assessed, a two-step system Politip for standardizing the polishing protocol was used to evaluate the surface roughness. The polishing procedure in our study was performed immediately after light curing. This recommendation made by Yap et al. is based on the fact that hygroscopic expansion will improve marginal adaptation by closing the gap formed by polymerization shrinkage and finishing/polishing procedures [30]. Venturini et al. proved that immediate polishing did not produce negative impact on the surface roughness, microhardness, and microleakage of novel RBCs compared to delayed polishing [31]. Contrary to this recommendation, there are authors that suggest to perform polishing procedures after 24 h when most part of RBC is polymerized [32]. Most clinicians do the polishing and finishing step immediately, which is more acceptable and cost effective for the patient.

The study examined the surface structure of four conventional composite materials and four bulk fill RBCs (Table 1). Similar Ra values of 0.20-0.26 *μ*m were obtained for four conventional nanohybrid materials. Despite the diverse structure of the organic and inorganic matrix, these materials have similar filler particle sizes, ranging from 0.01 to 2.0-3.0 *μ*m. As a result, these materials usually obtain similar and clinically acceptable Ra values. In the bulk fill material group, however, different surface roughness values ranging from 0.2 to 1.5 *μ*m were obtained. Bulk fill materials which have irregular particles >20 *μ*m in their structure (QF and XF) obtained several times higher Ra values than the other two bulk fill materials—TBF and FBF. A similar result was obtained by Costa et al. [33]. In this study, these materials also achieved higher roughness values compared to FBF and TBF. In turn, Parasher et al. obtained better surface profile properties for XF compared to TBF [34], but using the Shofu SuperSnap discs polishing system. In our research, we also compared the roughness of bulk fill materials: XF and QF with conventional GR and CX materials, and we obtained a several times higher roughness value for bulk fill materials. This result is consistent with the research of Costa et al., which showed a greater surface roughness after polishing with a three-stage Astropol system (Ivoclar Vivadent, New York) of a bulk fill X-tra fil composite compared to conventional GrandioSO material [33]. In turn, in our study, FBF obtained the lowest roughness of all bulk fill composites. FBF roughness was the same as another 3M-ESPE material—FZ. We obtained a similar result as Costa et al. in which in this study, FBF roughness after finishing and polishing was the same as that conventional Tetric N-Ceram composite and even increased after using additional polishing agent Astrobrush (Ivoclar Vivadent, New York) [30]. The results of this study suggest that not the type of material but the properties of its internal structure are the factors determining the roughness after polishing. This may be due to the particle size of the inorganic filler material FBF, which among the tested bulk fill materials has the smallest sizes. In addition to quantifying the surface structure of composite materials by profilometry, our studies also performed qualitative tests using the SEM imaging method. The qualitative assessment of the surface of materials in the SEM study, in most cases, corresponded well with the obtained values of material surface roughness.

The surface quality of conventional RBCs corresponded to a smooth surface, without damage—for TEC material and a smooth surface with slight damage—for GD, FZ, and CX (Table 3). In the TEC structure, the smallest filler particles are color pigments that have a diameter of 10 to 70 nm. Spherical oxide particles with a grain size of 180 nm are responsible for the mechanical properties, appropriate consistency of the material, and the chameleon effect due to their translucency and opalescence. The largest particles are 700 nm prepolymers made of monomers, ceramic fillers, and ytterbium fluoride. The SEM image of TEC only showed lines after polishing, without loss of filler particles. This observation is in contradiction to the data presented by Ehrmann et al. who obtained the loss of prepolymers after polishing, leaving irregularities on the surface of the material [5].

A similar SEM image of the surface was shown by conventional materials: GD and FZ. GD in its composition contains irregular filler particles with a diameter of about 1 *μ*m and silicon dioxide with a diameter of 20-40 nm, which agrees with the SEM image obtained in this study. In FZ, silica particles with a modified surface and 20 nm size occupy a significant part of the filler for better connection with the organic resin. The rest are modified zirconia particles and combined both types of particles into agglomerates with an average size of 3 *μ*m. The surface image of both materials showed that only small nanoparticles were torn out during polishing, without affecting the qualitative assessment of the smoothness of these materials. This agrees with the observations of Yap et al. [35] and Antonson et al. [36].

According to the manufacturer, the CX material contains organically modified nanoparticles with irregular structure in addition to standard 1 *μ*m filler particles. Those nanoparticles thanks to their 2.3 nm size allow it to obtain a homogeneous surface in the SEM image. In SEM images obtained in these studies, single defects of large particles are visible on the homogeneous surface of the material. The Ceram X composites have wider-diameter fillers than the other nanocomposites studied, which could also make them easier to rip out. However, this did not affect the obtained surface roughness values of the material. A similar result was obtained by Ehrmann et al. [5]. In our study, nanohybrid conventional composites were a group of materials that obtained similar surface quality after polishing with a two-step polishing system. In contrast, the SEM images of the bulk fill composite surface showed a variety of structures. The SEM image of the surface of the tested bulk fill materials showed that each of the tested materials has a different size and arrangement of inorganic fillers. Tetric Evoceram Bulk Fill had a similar structure as the conventional equivalent Tetric Evoceram. In TBF, the prepolymers occupy 17 wt. material by reducing polymerization shrinkage and increasing mechanical properties. The described construction of fillers corresponds to the obtained image in SEM. The qualitative assessment of the surface roughness of both of these materials was similar: TEC—smooth surface, no damage; TBF—smooth surface with slight damage. Tetric EvoCeram Bulk Fill was the only bulk fill material tested in the study that obtained a fairly good qualitative description of the surface after polishing (Table 3). TBF received a worse result of surface roughness after polishing in the Ehrmann et al. study [5].

According to the manufacturer, FBF has a very similar inorganic part structure to FZ. The difference in the inorganic part of FBF is the presence of 100 nm ytterbium trifluoride agglomerates to increase the contrast in X-rays. However, the SEM image of the FBF surface was assessed as a rough surface in contrast to the FZ material, which was rated as a smooth surface (Table 3). Loss of larger filler particles was responsible for this. Although it did not affect the quantitative assessment of the surface roughness of the material (Table 2), it significantly deteriorated the qualitative assessment, because in clinical conditions, they can be a source of discoloration of the material's surface.

Different appearance of the surface in SEM images from all tested materials was demonstrated by XF and QF bulk fill composites. XF is a multihybrid composite in which the inorganic part consists of barium-boron-aluminum-silicon glass particles. It contains in its structure both 40 and 100 nm particles as well as large, irregular particles exceeding 20 *μ*m in size. QF has a similar structure of the inorganic part as XF, which is composed of two sizes of irregular strontium-aluminum glass particles: smaller particles, 0.1-2.0 *μ*m; larger, 5-30 *μ*m. These materials, after applying the two-step polishing system, obtained a qualitative assessment—rough surface. In previous studies, Parasher et al. and Costa et al. achieved a greater roughness of these materials compared to other composite materials regardless of the polishing system used [33, 34]. These reports confirm the results of our research.

Results received in our quantitative and qualitative studies confirmed the H01 hypothesis with respect to conventional composites. It did not find confirmation in relation to bulk fill materials. The obtained results indicate that regular viscosity bulk fill composite materials constitute a group of materials diversified in terms of quantitative and qualitative assessment of surface roughness obtained after polishing. Also, compared to the conventional materials tested, they achieve, for the most part, poorer smoothness values after polishing, which allows the H02 hypothesis to be rejected. It can be assumed that this is due to the different and varied sizes of filler particles used in these materials. The obtained results confirm that the shape and size of filler particles seem to be of the greatest importance in determining the roughness of a composite material.

## 5. Conclusions

All tested composite materials which are recommended as posterior tooth fillings have different structures of both organic and inorganic matrix. Regular viscosity bulk fill composites turned out to be a diverse group of resin-based materials. They differ in their inorganic fillers from macro- to micro- to nanofillers, the size of the particles, and the extent of the filler loading in addition to the difference in the resin matrix. These factors influence their polishability. Regular viscosity bulk fill composites did not constitute a homogeneous group regarding surface roughness after polishing, presenting from smooth to rough surfaces, in contrast to conventional RBCs whose surfaces presented similar roughness after applying a uniform polishing system. The achieved results may indicate the need for individual selection of the appropriate technique and tool for polishing the surface of a regular viscosity bulk fill RBC in order to obtain optimal clinical appearance and properties of material used.

## Figures and Tables

**Table 1:** Materials used in the study and their composition.

Composite type	Material	Abbreviation	Manufacturer	Organic matrix	Inorganic filler	Filler content by weight (%)	Filler particle size (*μ*m)
Conventional	Tetric EvoCeram®	TEC	Ivoclar Vivadent (Schaan, Liechtenstein)	Bis-GMA, UDMA, Bis-EMA	Barium glass filler, Ytterbium trifluoride, mixed oxide	79.5	0.04-3
	GrandioSO®	GD	VOCO (Cuxhaven, Germany)	Bis-GMA, TEGDMA, Bis-EMA	Glass ceramic filler, silicon dioxide, pigments (iron oxide, titanium dioxide)	89.5	0.02-1
	Filtek™ Z550	FZ	3M-Espe (St. Paul, USA)	Bis-GMA, UDMA, Bis-EMA, PEGDMA, TEGDMA	Surface-modified zirconium dioxide/silica, surface-modified silica particles	82	0.02-3
	Ceram·X™ Mono	CX	Dentsply (Konstanz, Germany)	Methacrylate modified polysiloxane, Bis-GMA	Barium aluminum boron silicon glass, silicon oxide as a nanofiller particle	76	0.01-1.5
Regular viscosity bulk fill	Tetric EvoCeram® Bulk Fill	TBF	Ivoclar Vivadent (Schaan, Liechtenstein)	Bis-GMA, UDMA, Bis-EMA	Barium aluminium silicate glass, Isofiller, ytterbium fluoride, spherical mixed oxide	62.5 + 17 Isofiller	0.04-3
	Xtra-fil	XF	VOCO (Cuxhaven, Germany)	Bis-GMA, UDMA, TEGDMA	Anorganic fillers (no accurate data)	86	>20
	Filtek™ Bulk Fill Posterior	FBF	3M-Espe (St. Paul, USA)	AUDMA, AFM, DDDMA, UDMA	Nonagglomerated/nonaggregated silica filler, nonagglomerated/nonaggregated zirconia filler, aggregated zirconia/silica cluster filler, ytterbium trifluoride	76.5	0.004-0.1
	QuixFil™	QF	Dentsply (Konstanz, Germany)	UDMA, TEGDMA, di- and trimethacrylate resins, carboxylic acid-modified methacrylate resins	Silanized strontium-aluminum glass with the addition of sodium fluoride	86	0.15-30

Bis-GMA: bisphenol A−glycidyl methacrylate; UDMA: urethane dimethacrylate; Bis-EMA: ethoxylated bisphenol-A dimethacrylate; TEGDMA: triethylene glycol dimethacrylate; PEGDMA: poly (ethylene glycol) dimethacrylate; AUDMA: aromatic urethane dimethacylate; AFM: addition-fragmentation monomers; DDDMA: 1, 12-dodecanediol dimethacrylate.

**Table 2:** Average values of surface roughness of tested materials after polishing. Means with the same superscript symbol show statistically significant (*p* < 0.05) differences. The other means do not differ significantly.

Conventional composite	Roughness Ra (*μ*m)	Bulk fill composite	Roughness Ra (*μ*m)
	Mean ± SD		Mean ± SD
TEC	0.24 ± 0.15^f^	TBF	0.49 ± 0.10^A,B,C,g,i^
GD	0.22 ± 0.09^g^	XF	1.34 ± 0.42^A,D,f,g,h,i^
FZ	0.26 ± 0.10^h^	FBF	0.23 ± 0.07^B,D,E^
CX	0.20 ± 0.05^i^	QF	1.36 ± 0.14^C,E,f,g,h,i^

**Table 3:** Qualitative assessment of the materials surfaces in the SEM study. The difference in images assessment between the two researchers was not greater than 10%.

Conventional composite	Spot SEM evaluation of the surface	Descriptive evaluation of the surface	Bulk fill composite	Spot SEM evaluation of the surface	Descriptive evaluation of the surface
	Mean			Mean	
TEC	10.5	Smooth surface, no damage	TBF	12.5	Smooth surface with slight damage
GD	22.5	Smooth surface with slight damage	XF	35.5	Rough surface
FZ	19.5	Smooth surface with slight damage	FBF	31	Rough surface
CX	19.5	Smooth surface with slight damage	QF	34.5	Rough surface

## Data Availability

The data that support the findings of this study are available on request from the corresponding author, J. C, U.K. The data are not publicly available due to their containing information that could compromise the privacy of research participants.

## References

[1] Jefferies S. R. (2007). Abrasive finishing and polishing in restorative dentistry: a state-of-the-art review. *Dental Clinics of North America*.

[2] Marghalani H. Y. (2010). Effect of filler particles on surface roughness of experimental composite series. *Journal of Applied Oral Science*.

[3] Berastegui E., Canalda C., Brau E., Miquel C. (1992). Surface roughness of finished composite resins. *The Journal of Prosthetic Dentistry*.

[4] Barbosa S. H., Zanata R. L., de Lima Navarro M. F., Nunes O. B. (2005). Effect of different finishing and polishing techniques on the surface roughness of microfilled, hybrid and packable composite resins. *Brazilian Dental Journal*.

[5] Ehrmann E., Medioni E., Brulat-Bouchard N. (2019). Finishing and polishing effects of multiblade burs on the surface texture of 5 resin composites: microhardness and roughness testing. *Restorative dentistry & endodontics*.

[6] Endo T., Finger W. J., Kanehira M., Utterodt A., Komatsu M. (2010). Surface texture and roughness of polished nanofill and nanohybrid resin composites. *Dental Materials Journal*.

[7] Yap A. U., Wu S. S., Chelvan S., Tan E. S. (2005). Effect of hygiene maintenance procedures on surface roughness of composite restoratives. *Operative Dentistry*.

[8] Alfawaz Y. (2017). Impact of polishing systems on the surface roughness and microhardness of nanocomposites. *The Journal of Contemporary Dental Practice*.

[9] Korkmaz Y., Ozel E., Attar N., Aksoy G. (2008). The influence of one-step polishing systems on the surface roughness and microhardness of nanocomposites. *Operative Dentistry*.

[10] St-Pierre L., Martel C., Crépeau H., Vargas M. A. (2019). Influence of polishing systems on surface roughness of composite resins: polishability of composite resins. *Operative Dentistry*.

[11] Ishii R., Takamizawa T., Tsujimoto A. (2020). Effects of finishing and polishing methods on the surface roughness and surface free energy of bulk-fill resin composites. *Operative Dentistry*.

[12] Arisu H. D., Üçtaşli M. B., Ömürlü H. (2007). The effect of different finishing and polishing systems on the surface roughness of different composite restorative materials. *The Journal of Contemporary Dental Practice*.

[13] Curtis A. R., Palin W. M., Fleming G. J. P., Shortall A. C. C., Marquis P. M. (2009). The mechanical properties of nanofilled resin-based composites: the impact of dry and wet cyclic pre-loading on bi-axial flexure strength. *Dental Materials*.

[14] Ferracane J. L. (2011). Resin composite--State of the art. *Dental Materials*.

[15] Jones C. S., Billington R. W., Pearson G. J. (2004). The in vivo perception of roughness of restorations. *British Dental Journal*.

[16] Weitman R. T., Eames W. B. (1975). Plaque accumulation on composite surfaces after various finishing procedures. *Journal of the American Dental Association (1939)*.

[17] Ruschel V. C., Bona V. S., Baratieri L. N., Maia H. P. (2018). Effect of surface sealants and polishing time on composite surface roughness and microhardness. *Operative Dentistry*.

[18] Bollen C. M., Lambrechts P., Quirynen M. (1997). Comparison of surface roughness of oral hard materials to the threshold surface roughness for bacterial plaque retention: a review of the literature. *Dental Materials*.

[19] Chung K. H. (1994). Effects of finishing and polishing procedures on the surface texture of resin composites. *Dental Materials*.

[20] Willems G., Lambrechts P., Braem M., Vuylsteke-Wauters M., Vanherle G. (2016). The surface roughness of enamel-to-enamel contact areas compared with the intrinsic roughness of dental resin composites. *Journal of Dental Research*.

[21] Shintani H., Satou J., Satou N., Hayashihara H., Inoue T. (1985). Effects of various finishing methods on staining and accumulation of Streptococcus mutans HS-6 on composite resins. *Dental Materials*.

[22] Sahbaz C., Bahsi E., Ince B., Bakir E. P., Cellik O. (2016). Effect of the different finishing and polishing procedures on the surface roughness of three different posterior composite resins. *Scanning*.

[23] Van Ende A., De Munck J., Lise D. P., Van Meerbeek B. (2017). Bulk-fill composites: a review of the current literature. *The Journal of Adhesive Dentistry*.

[24] Łagocka R., Jakubowska K., Chlubek D., Buczkowska-Radlińska J. (2016). The influence of irradiation time and layer thickness on elution of triethylene glycol dimethacrylate from SDR® bulk-fill composite. *Biomed Research International*.

[25] Leprince J. G., Palin W. M., Vanacker J., Sabbagh J., Devaux J., Leloup G. (2014). Physico-mechanical characteristics of commercially available bulk-fill composites. *Journal of Dentistry*.

[26] ISO E. N. (2009). 4049: 2009 Dentistry-Polymer-based restorative materials. *ISO International Organization for Standardization*.

[27] Pereira C. A., Eskelson E., Cavalli V., Liporoni P. C. S., Jorge A. O. C., Rego M. A. . (2011). Streptococcus mutans biofilm adhesion on composite resin surfaces after different finishing and polishing techniques. *Operative Dentistry*.

[28] Gönülol N., Yilmaz F. (2012). The effects of finishing and polishing techniques on surface roughness and color stability of nanocomposites. *Journal of Dentistry*.

[29] Stoddard J. W., Johnson G. H. (1991). An evaluation of polishing agents for composite resins. *The Journal of Prosthetic Dentistry*.

[30] Yap A. U., Ang H. Q., Chong K. C. (2009). Influence of finishing time on marginal sealing ability of new generation composite bonding systems. *Journal of Oral Rehabilitation*.

[31] Venturini D., Cenci M. S., Demarco F. F., Camacho G. B., Powers J. M. (2006). Effect of polishing techniques and time on surface roughness, hardness and microleakage of resin composite restorations. *Operative Dentistry*.

[32] Madhyastha P. S., Hegde S., Srikant N., Kotian R., Iyer S. S. (2017). Effect of finishing/polishing techniques and time on surface roughness of esthetic restorative materials. *Dent Res J*.

[33] de Fátima Alves da Costa G., Melo A. M. D. S., de Assunção I. V., Borges B. C. D. (2020). Impact of additional polishing method on physical, micromorphological, and microtopographical properties of conventional composites and bulk fill. *Microscopy Research and Technique*.

[34] Parasher A., Ginjupalli K., Somayaji K., Kabbinale P. (2020). Comparative evaluation of the depth of cure and surface roughness of bulk-fill composites: an in vitro study. *Dent Med Probl.*.

[35] Yap A. U., Tan C. H., Chung S. M. (2004). Wear behavior of new composite restoratives. *Operative Dentistry*.

[36] Antonson S. A., Yazici A. R., Kilinc E., Antonson D. E., Hardigan P. C. (2011). Comparison of different finishing/polishing systems on surface roughness and gloss of resin composites. *Journal of Dentistry*.

